# Supplementary material for: Multi-layered transcriptional control of glycogen metabolism coordinates thermogenic remodeling of white adipocytes in male mice
Source: Nat Commun. 2025 Dec 16;17:809. doi: 10.1038/s41467-025-67515-9 (PMC12824187; doi:10.1038/s41467-025-67515-9)

Supplementary Fig.1

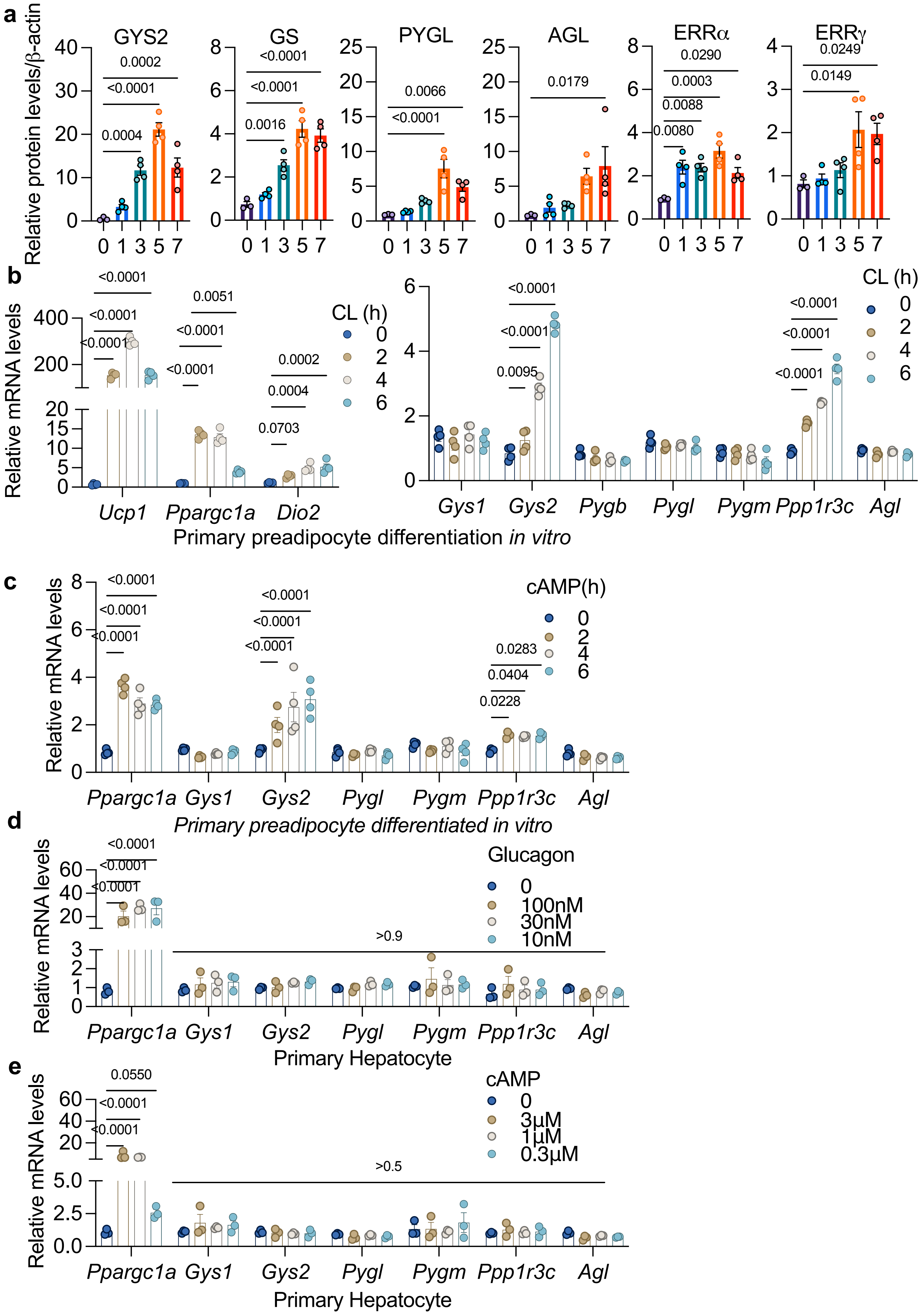

Supplementary Fig. 1 | β3-adrenergic receptor activation regulates glycogen metabolism genes in a cell-autonomous manner.

a. Quantification of proteins in **Fig.1b**. *P* values was determined by two-sided one-way ANOVA followed by Dunnett's multiple comparisons test.

b-c. Gene expression of glycogen metabolism and thermogenic genes in primary preadipocytes differentiated *in vitro* and treated with CL316,243(**b**) or cell-permeable cAMP (**c**) for the indicated durations. *n* = 4 biological replicates per treatment.

d. Gene expression in wild-type primary hepatocytes treated with varying doses of glucagon. *n* = 3 biological replicates per treatment.

e. Gene expression in wild-type primary hepatocytes treated with increasing concentrations of cell-permeable cAMP. *n* = 3 biological replicates per treatment.

Data (**b** - **e**) show mean ± s.e.m., *P* values were determined by two-sided two-way ANOVA followed by Dunnett's multiple comparisons test.

Supplementary Fig.2

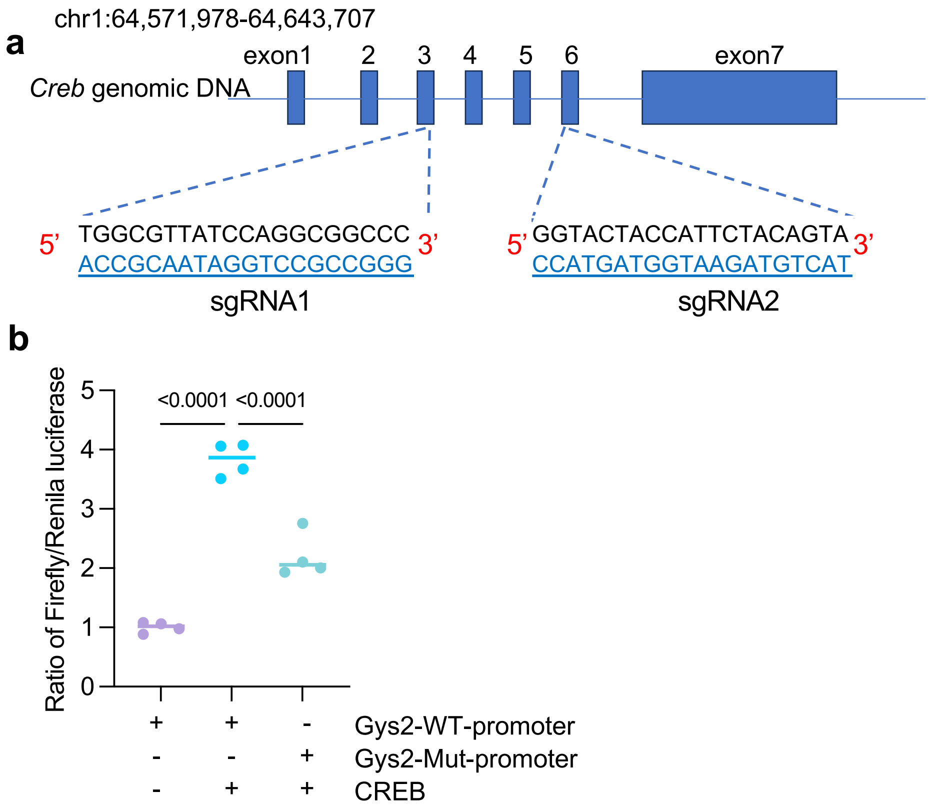

Supplementary Fig. 2 | CREB directly regulates *Gys2* expression downstream of PKA signaling.

a. Schematic of the *Creb* genomic locus showing sgRNA1 and sgRNA2 targeting sites used for CRISPR editing. Exons are indicated as blue boxes. Target sequences (blue) with PAM sites (underlined) are shown.

b. Luciferase reporter assay in HEK293T cell transfected with wild-type (WT) or CRE-binding site–mutated (Mut) *Gys2* promoter constructs, with or without CREB overexpression. Firefly luciferase activity was normalized to Renilla luciferase, and results are shown as ratios. (n = 4 biological replicates). *P* values were determined by two-sided one-way ANOVA followed by Tukey's multiple comparisons test.

Supplementary Fig.3

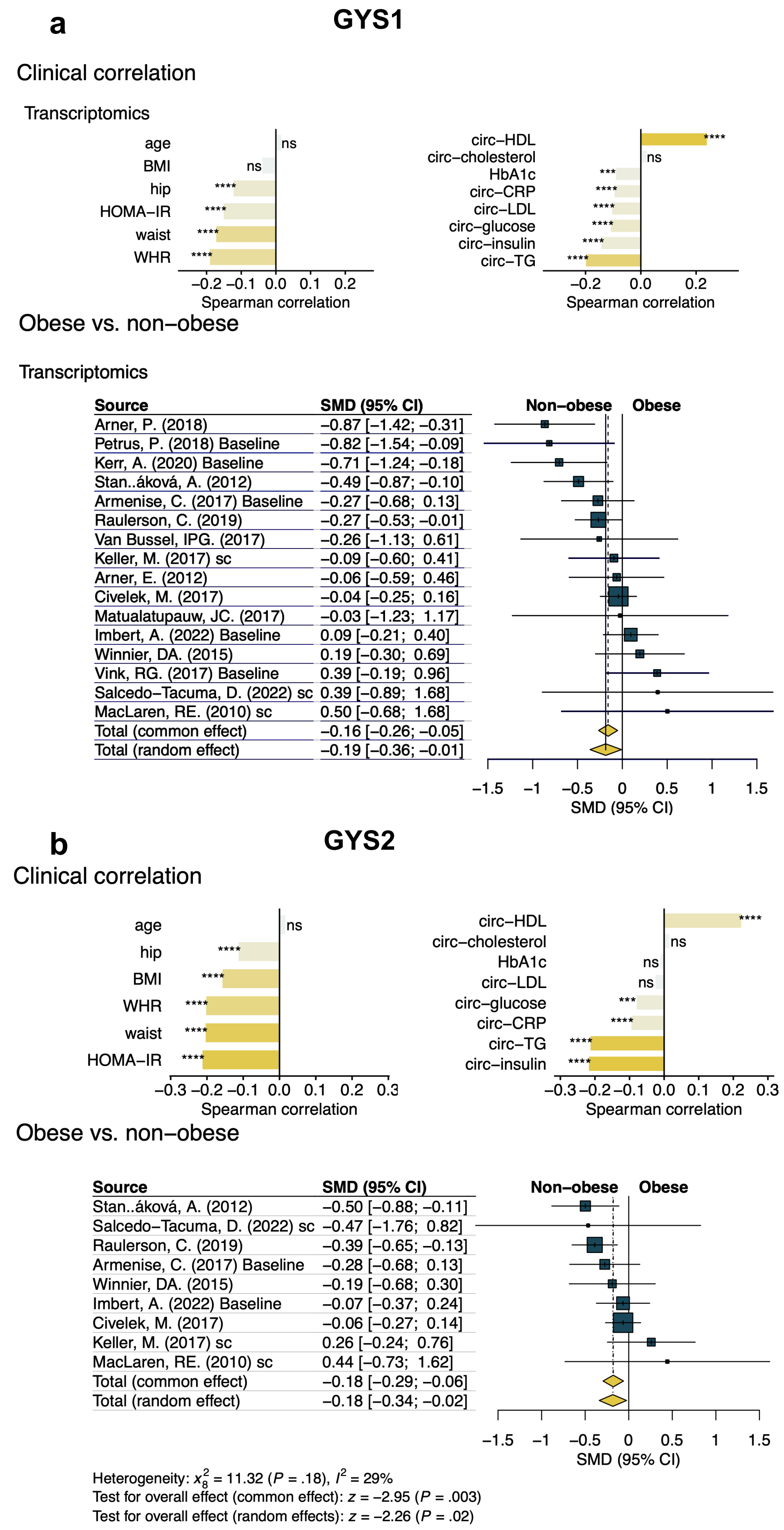

Supplementary Fig. 3 | Association of GYS1 and GYS2 with metabolic traits in human studies.

a, b. Data adapted from the Adipose Tissue Knowledge Portal showing associations of *Gys1* and *Gys2* with clinical and/or adipocyte-related traits in cross-sectional and longitudinal cohorts.

Supplementary Fig.4

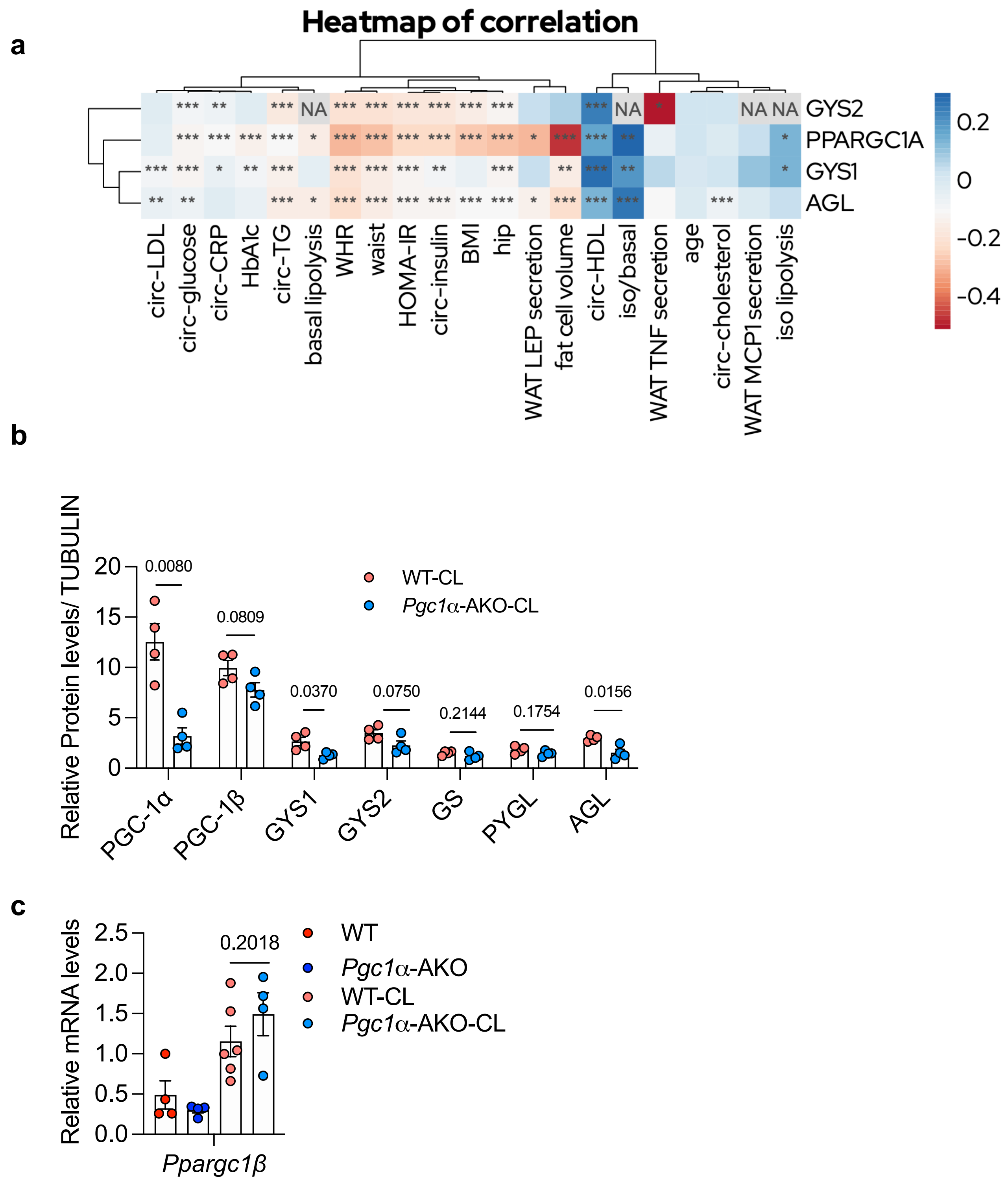

Supplementary Fig. 4 | Correlation of glycogen metabolism gene expression and *PPARGC1A*.
a. Phenotype correlation of gene expression for GYS1, GYS2, AGL, and PPARGC1A in multiple clinical cohorts and trait, Data adapted from the Adipose Tissue Knowledge Portal.
b. Quantification of protein in **Fig.3b.**
c. *Ppargc1b mRNA levels* in iWAT of mice treated as in **Fig.3c**.
Data **b** and **c** show mean ± s.e.m., *P* values were determined by unpaired t test with Welch correction

Supplementary Fig.5

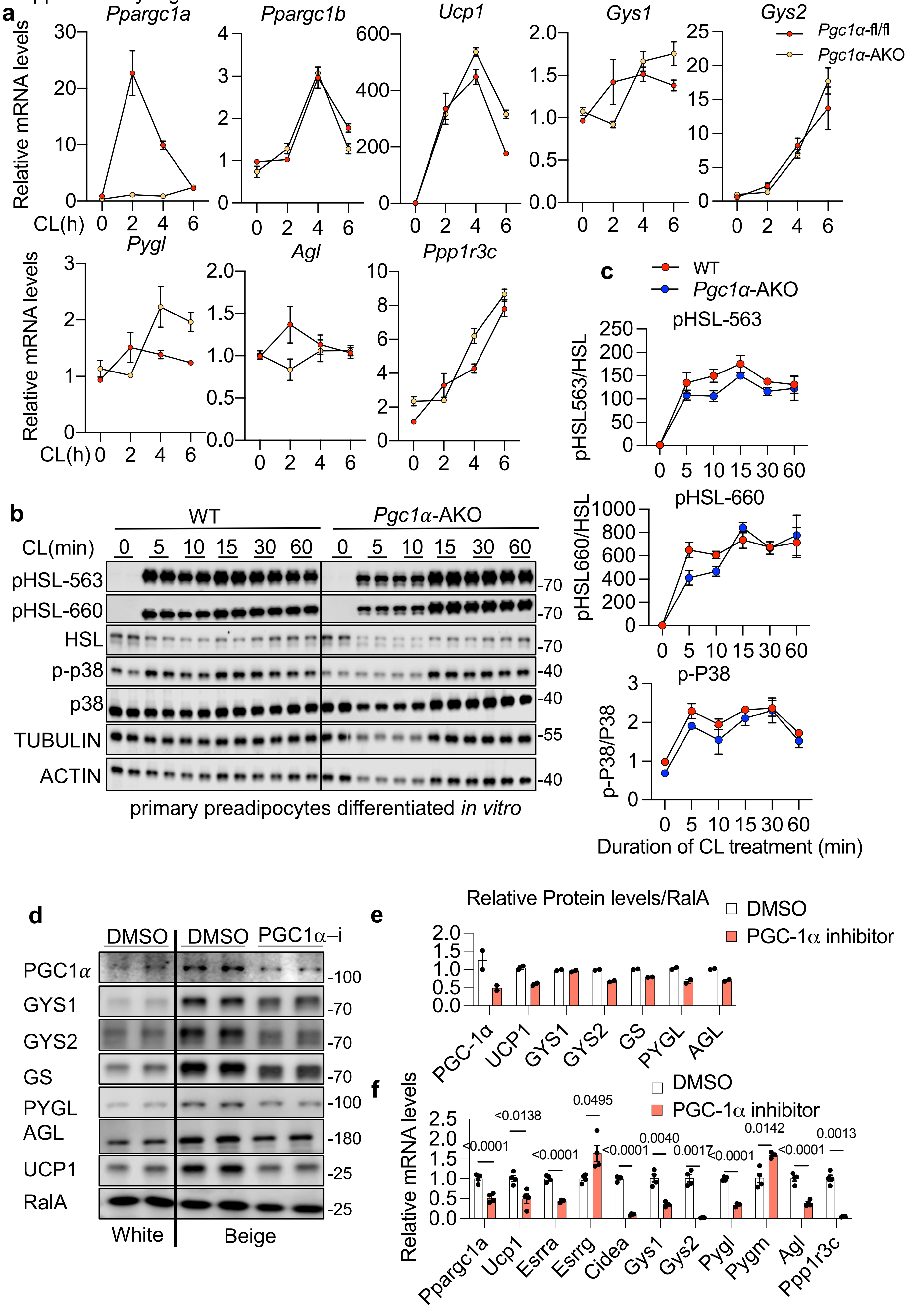

Supplementary Fig. 5 | PGC1α sustains glycogen metabolism gene expression during chronic β3-adrenergic activation.

a. Gene expression in wild-type (WT) and PGC1α-adipocyte knockout (PGC1α-AKO) primary preadipocytes differentiated *in vitro* after the indicated durations of CL-316,243 treatment. *n* = 4 biological replicates per genotype per treatment.

b. Activation of p38 and HSL phosphorylation by CL-316,243 in WT and PGC1α-AKO primary preadipocytes differentiated *in vitro*. *n* = 2 biological replicates per genotype per treatment.

c. Quantification of protein levels in **b.**

d. Protein expression in primary white and beige preadipocytes differentiated *in vitro* and treated with either DMSO or PGC1α inhibitor (10 μM SR-18292) for 12 hours. *n* = 2 biological replicates per genotype per treatment.

e. Quantification of protein levels in beige adipocyte differentiated *in vitro* treated with either DMSO or PGC1α inhibitor**.**

f. Gene expression in beige adipocytes differentiated *in vitro* and treated with either DMSO or PGC1α inhibitor (10 μM SR-18292) for 12 hours. *n* = 4 biological replicates per treatment.

Data **f** shows mean ± s.e.m., *P* values were determined by unpaired t test with Welch correction

Supplementary Fig.6

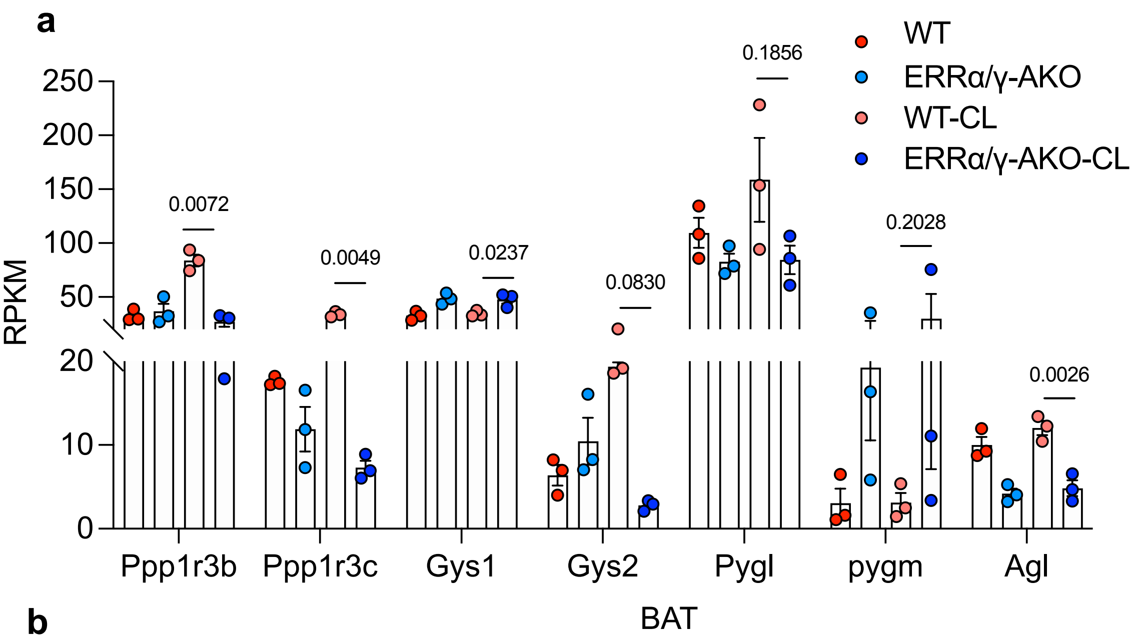

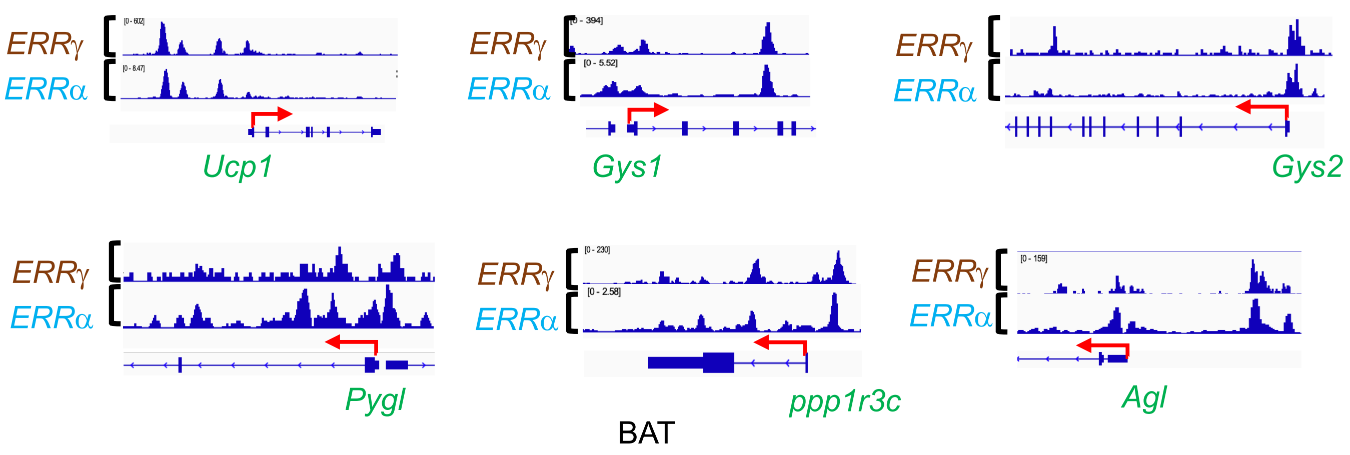

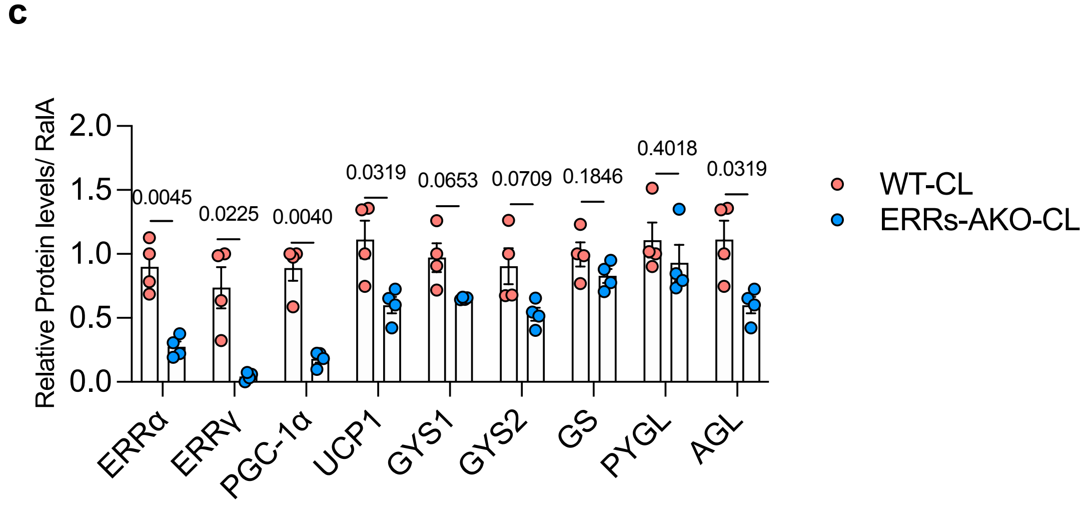

Supplementary Fig. 6 | ERRα/γ maintain glycogen metabolism genes expression during chronic β3-adrenergic signaling in BAT

a. RPKM values of indicated genes in brown adipose tissue (BAT) of wild-type (WT) and ERRα/γ adipose-specific knockout (ERRα/γ-AKO) mice treated with CL-316,243 for 10 days, adapted from GSE104285.

b. ChIP–seq analysis showing binding of ERRα (GSE83928) and ERR γ (SRP063705) at selected gene loci in BAT. Tracks indicate normalized ChIP–seq signal density centered on transcriptional start sites of target genes.

c. Quantification of protein levels in **Fig.4a.**

Data **a** and **c** show mean ± s.e.m., *P* values were determined by a two-sided unpaired *t*-test with Welch's correction.

Supplementary Fig.7

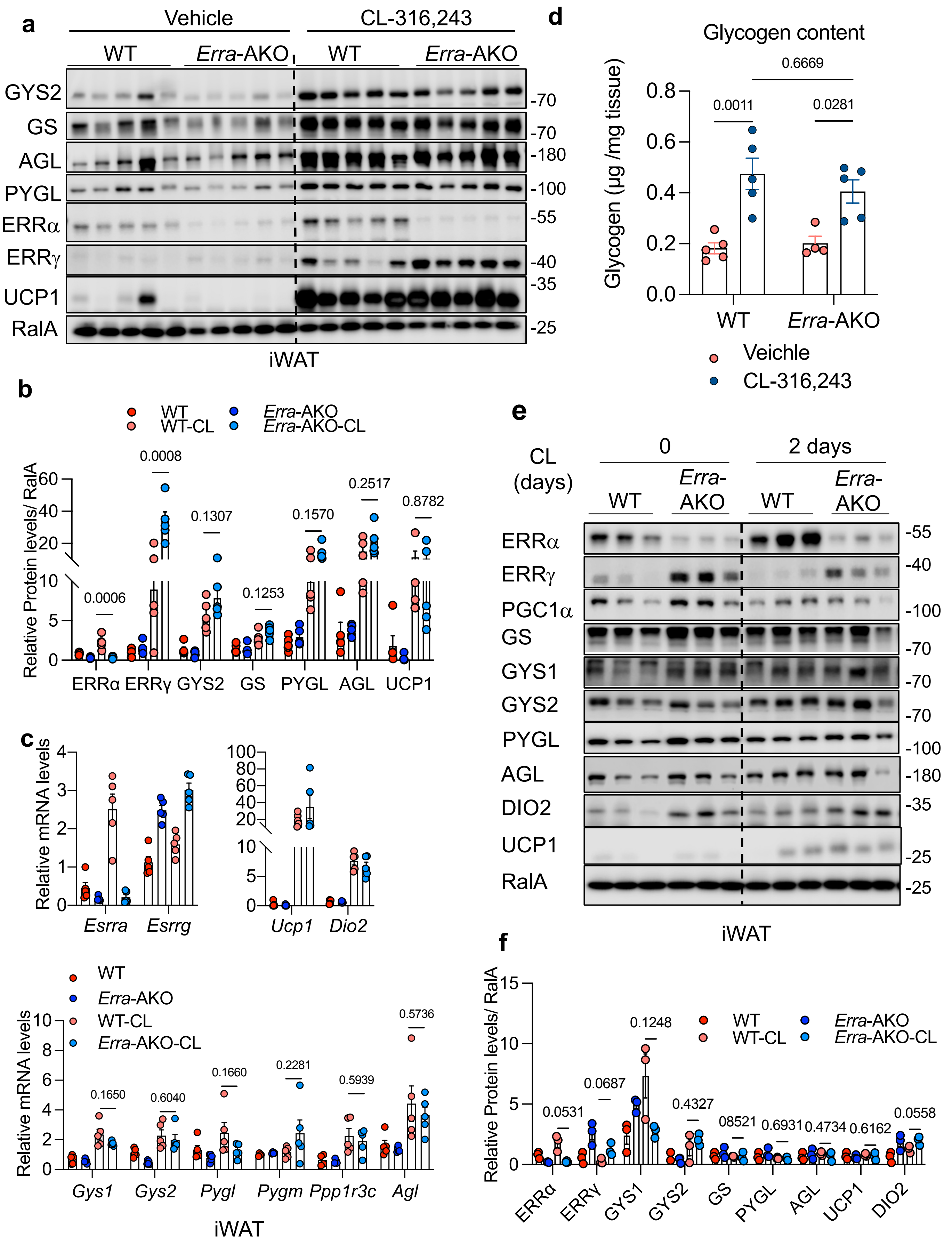

Supplementary Fig. 7 | ERRγ shows increased expression in iWAT of ERRα-AKO mice

a. Protein expression in inguinal white adipose tissue (iWAT) from wild-type (WT) and ERRα-AKO mice treated with either vehicle or CL-316,243 (1 mg/kg/day) for 7 days. *n* = 5 mice per genotype per treatment.

b. Quantification of protein levels in **a.**

c. Gene expression in iWAT of WT and ERRα-AKO mice treated with either vehicle or CL-316,243 for 7 days. Gene expression was quantified by RT-qPCR. *n* = 5 mice per treatment per genotype.

d. Glycogen levels in iWAT from WT and ERRα-AKO mice treated with either vehicle or CL-316,243 for 2 days. Glycogen content was determined using a glycogen assay kit. *n* = 3 mice per treatment per genotype.

e. Protein expression in iWAT of WT and ERRα-AKO mice treated with either vehicle or CL-316,243 (1 mg/kg/day) for 7 days. Data are presented as *n* = 4 - 5 mice per treatment per genotype.

f. Quantification of protein levels in **e.**

Data **d** shows mean ± s.e.m., *P* values were determined by two-sided Ordinary one-way ANOVA followed by Tukey's multiple comparisons test

Data **b** shows mean ± s.e.m., *P* values were determined by Unpaired t test with Welch correction

Supplementary Fig.8

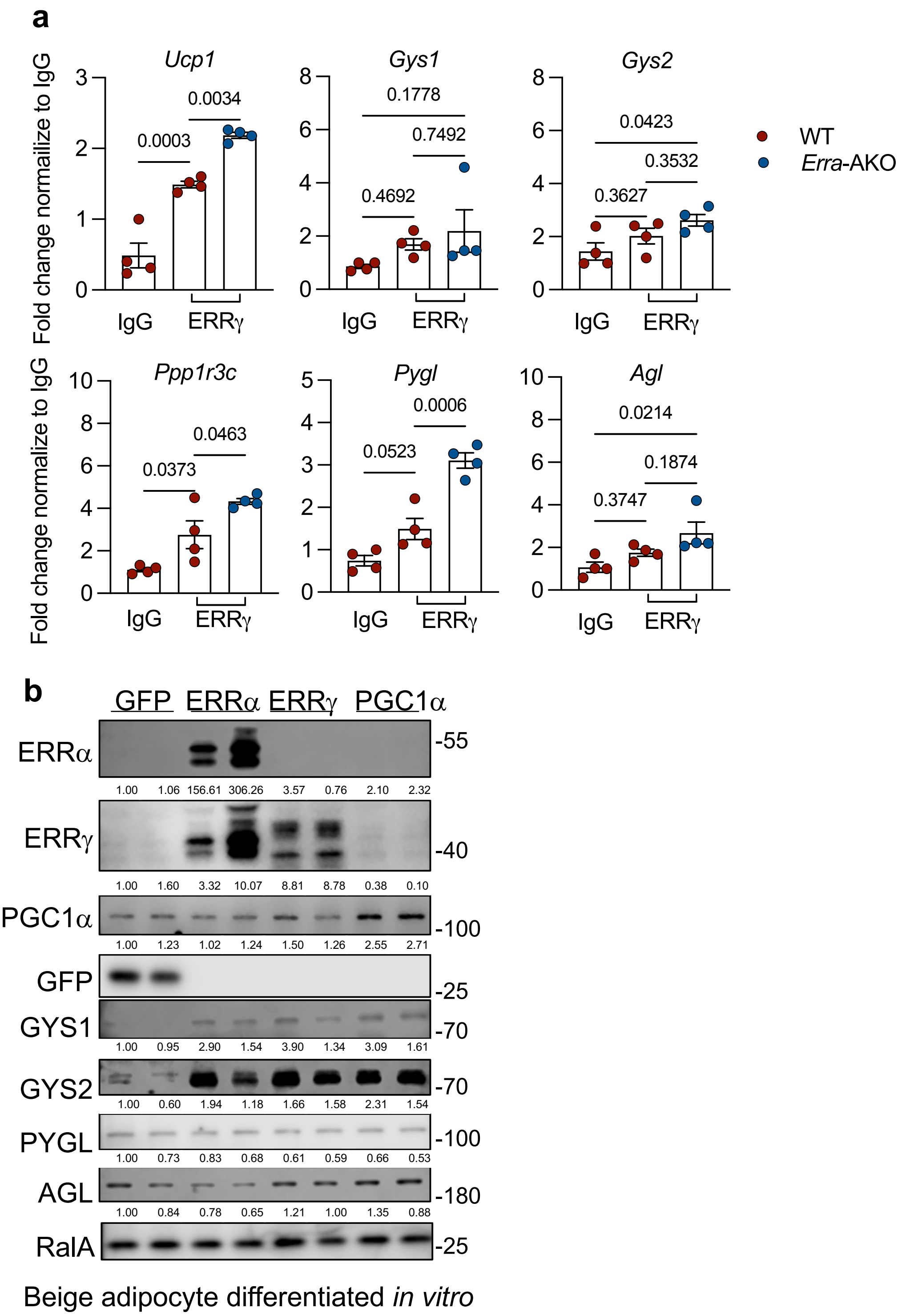

Supplementary Fig.8| ERRγ shows increased binding at ERRα target sites in iWAT of ERRα-AKO mice

a. ChIP–qPCR assay using an antibody against ERRγ in iWAT from WT and Erra-AKO mice treated for 7 days CL. *n* = 4 mice per genotype per treatment.Data show mean ± s.e.m., *P* values were determined by One-way ANOVA followed by Tukey's multiple comparisons test.

b. Protein expression in beige preadipocytes differentiated in vitro, overexpressing GFP, ERRα,ERR γ, and PGC1α. *n* = 2 biological replicates per genotype per treatment.

Supplementary Fig.9

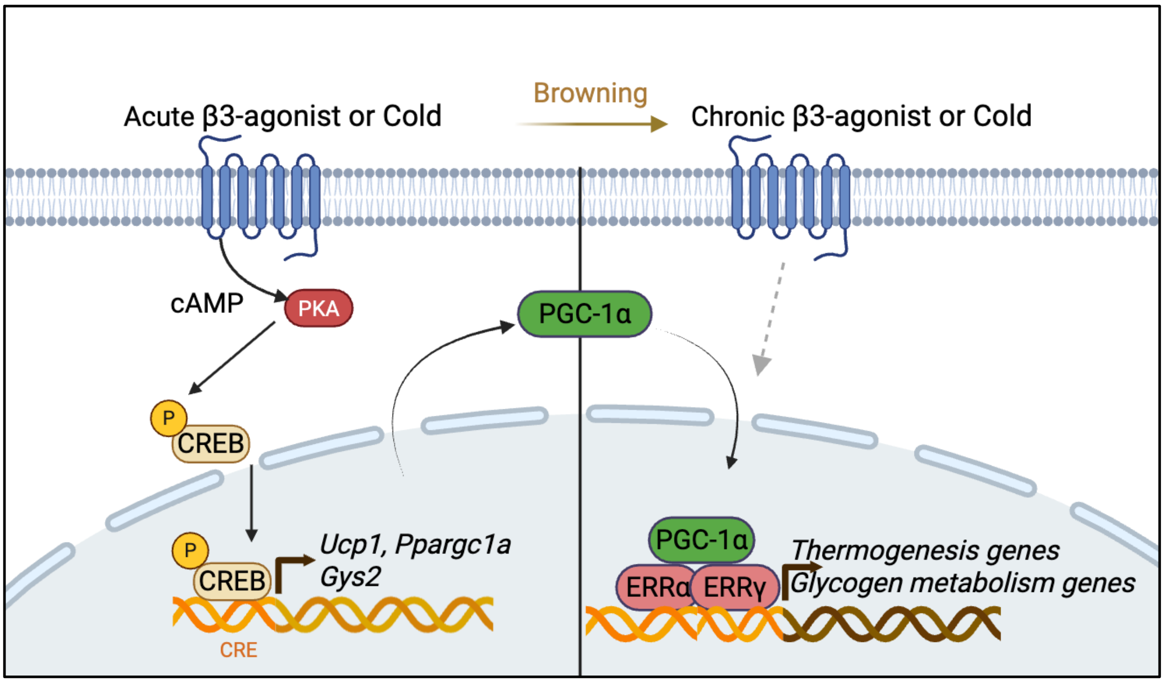

Supplementary Fig.9. Mechanistic model.
In inguinal white adipose tissue, acute β3-adrenergic activation engages CREB to prime the expression of *Gys2*. With chronic stimulation, PGC1α cooperates with ERRα and ERRγ, to maintain the expression of glycogen metabolism genes. Created in BioRender. Fu, H. (2025) https://BioRender.com/rvv6vnd

Supplementary Table 1. qPCR primers used in this study

	Forward	Reverse
Ucp1	AGGCTTCCAGTACCATTAGGT	CTGAGTGAGGCAAAGCTGATTT
Dio2	AATTATGCCTCGGAGAAGACCG	GGCAGTTGCCTAGTGAAAGGT
Cidea	TGACATTCATGGGATTGCAGAC	GGCCAGTTGTGATGACTAAGAC
Gys1	ATCTACACTGTGCTGCAGACG	CCCTTGCTGTTCATGGAATCC
Gys2	CCATCCTCAGCACCATTAGAC	GTGACAACCTCGGACAAACTC
Pygl	TGCTTTGGATAAGAAGGGGTATGAGGC	TTGAAGAGGTCTGGCTGATTGGGAG
Pygm	ATCAACCCCAACTCGCTCTTT	GCTCCCTTTTGATGCGGTT
Pygb	CAGCAGCATTACTATGAGCGG	CCAAGTCCAACCCCAACTGA
Esrra	TGCTCAGCTCTCTACCCAAAC	GGACAGCTGTACTCGATGCTC
Esrrb	CCGGCCACCAATGAATGT	ATCCAGCCGTCGCTTGTACT
Esrrg	ATGCCCAAGAGACTGTGCTT	CTTCTTTCAGCATGCCCACT
Agl	GTCCACAGATCGACCGAAACA	GTGCCACAGTTGAAGCGATTT
Ppargc1a	AGCCGTGACCACTGACAACGAG	GCTGCATGGTTCTGAGTGCTAAG
Ppargc1b	CTGCTGGCCCAGATACACTGA	ATCCATGGCTTCATACTTGCT
Creb	AGCCGGGTACTACCATTCTAC	GCAGCTTGAACAACAACTTGG
Atf2	AAGTCTGGCTATCATACTGCTGA	GCCATGACAATCTGTGAAAGTGC
36B4	AGATGCAGCAGATCCGCAT	GTTCTTGCCCATCAGCACC
Ucp1_ChIP	TTTTGTTCTTGCACTCACGCC	CCCATGGTGGGTTGCACTTC
pygl_ChIP	GGGCAATCAGGGTTAAATCA	TGTTGAGTTCCCAGTAAAGC
gys1_ChIP	GGCCTACAGCTGAGATTGAC	GCGTCTACTTATTGGCGGAT
gys2_ChIP	CCTGTAGAGGCTATCCCAA	GTTGCAAGTAGTACAAACCTT
Agl-ChIP	AGACCCGAATTTGGGAACTG	GGCCCTGAAAACTCCAAATG
Ptg-ChIP	TGGCTTTGATAAGCTGCCTC	TGAGCAACTTCGCACTCAG

Uncropped scans of all blots in Supplementary Figures

Supplementary Fig 5b

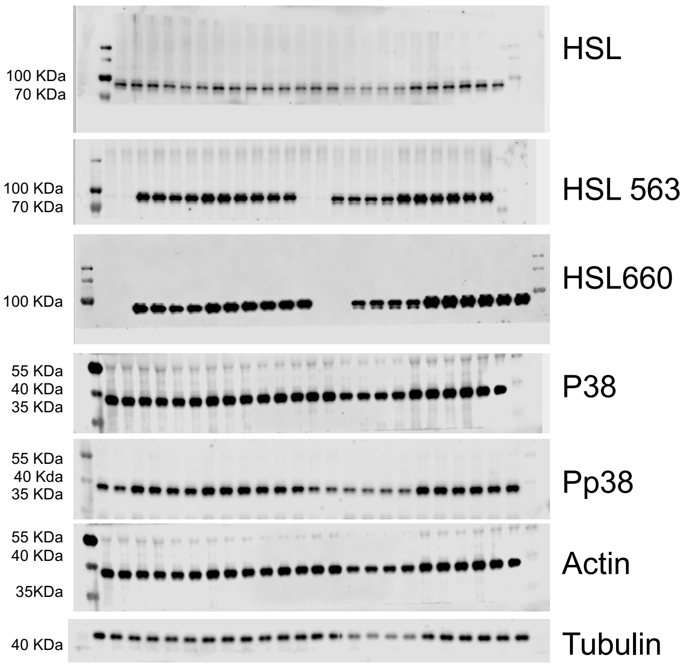

Supplementary Fig 5d

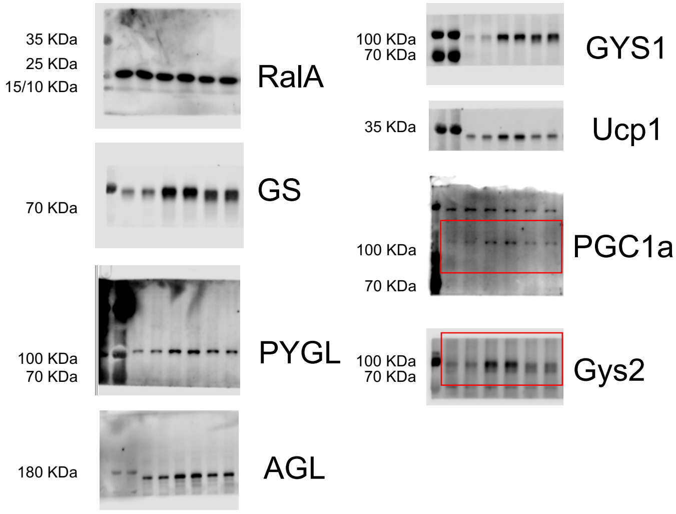

Supplementary Fig 7a

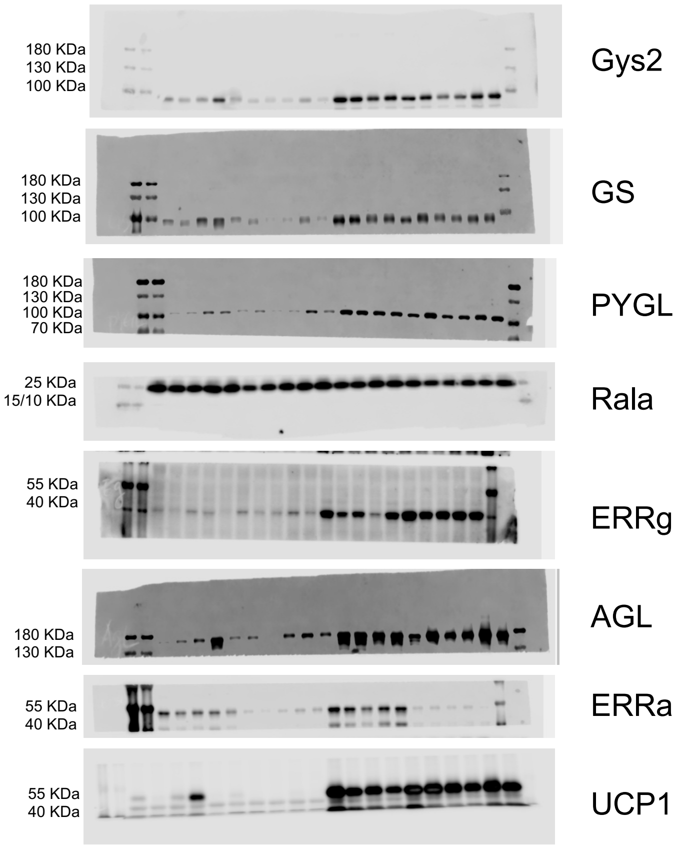

Supplementary Fig 7e

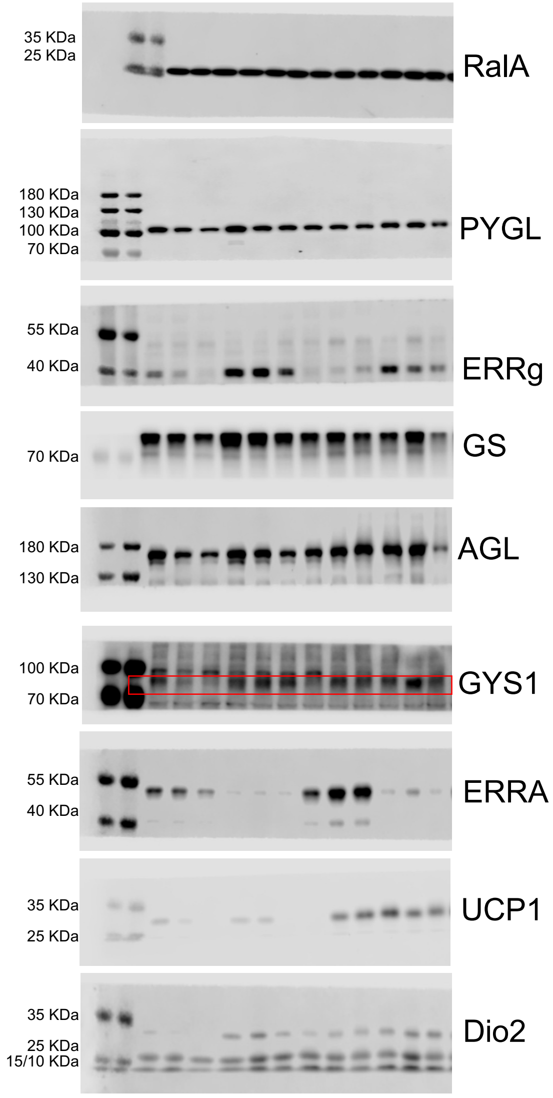

Uncropped scans of all blots in Supplementary Figures

Supplementary Fig 8b

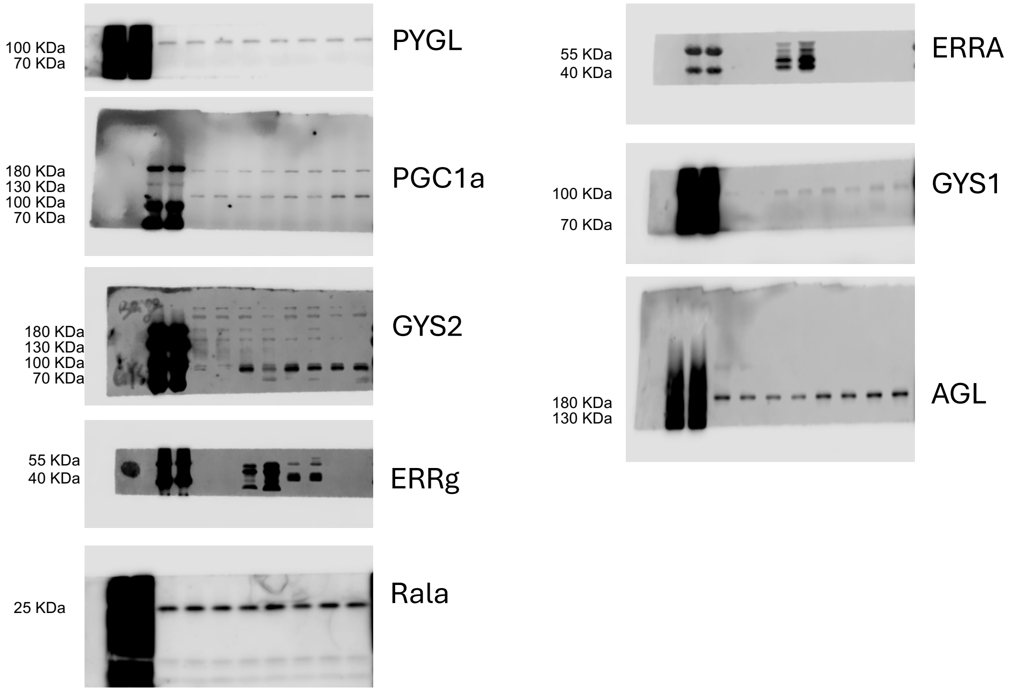

Supplement: Supplementary file 1 — Supplementary Information [file 41467_2025_67515_MOESM1_ESM.pdf]
