# Supplementary material for: Multi-layered transcriptional control of glycogen metabolism coordinates thermogenic remodeling of white adipocytes in male mice
Source: Nat Commun. 2025 Dec 16;17:809. doi: 10.1038/s41467-025-67515-9 (PMC12824187; doi:10.1038/s41467-025-67515-9)
Supplement: Supplementary file 2 — Reporting Summary [file 41467_2025_67515_MOESM2_ESM.pdf]

nature portfolio

Corresponding author(s): Alan Saltiel

Last updated by author(s): Nov 15, 2025

# Reporting Summary

Nature Portfolio wishes to improve the reproducibility of the work that we publish. This form provides structure for consistency and transparency in reporting. For further information on Nature Portfolio policies, see our Editorial Policies and the Editorial Policy Checklist.

## Statistics

For all statistical analyses, confirm that the following items are present in the figure legend, table legend, main text, or Methods section.

| n/a | Confirmed | |
|---|---|---|
| ☐ | ☒ | The exact sample size (*n*) for each experimental group/condition, given as a discrete number and unit of measurement |
| ☐ | ☒ | A statement on whether measurements were taken from distinct samples or whether the same sample was measured repeatedly |
| ☐ | ☒ | The statistical test(s) used AND whether they are one- or two-sided<br>*Only common tests should be described solely by name; describe more complex techniques in the Methods section.* |
| ☒ | ☐ | A description of all covariates tested |
| ☐ | ☒ | A description of any assumptions or corrections, such as tests of normality and adjustment for multiple comparisons |
| ☐ | ☒ | A full description of the statistical parameters including central tendency (e.g. means) or other basic estimates (e.g. regression coefficient) AND variation (e.g. standard deviation) or associated estimates of uncertainty (e.g. confidence intervals) |
| ☒ | ☐ | For null hypothesis testing, the test statistic (e.g. *F*, *t*, *r*) with confidence intervals, effect sizes, degrees of freedom and *P* value noted<br>*Give P values as exact values whenever suitable.* |
| ☒ | ☐ | For Bayesian analysis, information on the choice of priors and Markov chain Monte Carlo settings |
| ☒ | ☐ | For hierarchical and complex designs, identification of the appropriate level for tests and full reporting of outcomes |
| ☒ | ☐ | Estimates of effect sizes (e.g. Cohen's *d*, Pearson's *r*), indicating how they were calculated |

*Our web collection on statistics for biologists contains articles on many of the points above.*

## Software and code

Policy information about availability of computer code

| | |
|---|---|
| Data collection | NovaSeq X Plus Sequencing System ,QuantStudio™ 5 Real-Time PCR,LICORbio |
| Data analysis | Microsoft Excel 2016; GraphPad Prism V9 ; IGV_2.8.13 ; HiSeq Control (v3.4.0) Software;<br>Cufflinks v2.2.1 ;TopHat 2.0.6; RStudio (V1.2.1335);IMAGE STUDIO(Version 6.0);Picard (v2.26.10) |

For manuscripts utilizing custom algorithms or software that are central to the research but not yet described in published literature, software must be made available to editors and reviewers. We strongly encourage code deposition in a community repository (e.g. GitHub). See the Nature Portfolio guidelines for submitting code & software for further information.

## Data

Policy information about availability of data

All manuscripts must include a data availability statement. This statement should provide the following information, where applicable:

- Accession codes, unique identifiers, or web links for publicly available datasets
- A description of any restrictions on data availability
- For clinical datasets or third party data, please ensure that the statement adheres to our policy

*Provide your data availability statement here.*

## Research involving human participants, their data, or biological material

Policy information about studies with human participants or human data. See also policy information about sex, gender (identity/presentation), and sexual orientation and race, ethnicity and racism.

| | |
|---|---|
| Reporting on sex and gender | N/A |
| Reporting on race, ethnicity, or other socially relevant groupings | N/A |
| Population characteristics | N/A |
| Recruitment | N/A |
| Ethics oversight | N/A |

Note that full information on the approval of the study protocol must also be provided in the manuscript.

# Field-specific reporting

Please select the one below that is the best fit for your research. If you are not sure, read the appropriate sections before making your selection.

☒ Life sciences ☐ Behavioural & social sciences ☐ Ecological, evolutionary & environmental sciences

For a reference copy of the document with all sections, see nature.com/documents/nr-reporting-summary-flat.pdf

# Life sciences study design

All studies must disclose on these points even when the disclosure is negative.

| | |
|---|---|
| Sample size | No specific methods were used for sample size determination. Sample size for all the presented experiments was determined to be at least 3 to provide sufficient data for downstream analysis. |
| Data exclusions | No data were excluded |
| Replication | For each experiments the number of biological independent sample is reported in the figure legend. In vitro studies are represented at least 3 independent reproducible studies. |
| Randomization | Mice were randomly allocated into experimental groups and control groups. No randomization was necessary since all the in vitro experiments presented in this study were at least 3 independent experiments to confirm the results. All samples were treated side-by-side under the same conditions and compared to relevant controls. |
| Blinding | No blinding was necessary since all the in vitro experiments presented in this study were at least duplicated to confirm the results. |

# Reporting for specific materials, systems and methods

We require information from authors about some types of materials, experimental systems and methods used in many studies. Here, indicate whether each material, system or method listed is relevant to your study. If you are not sure if a list item applies to your research, read the appropriate section before selecting a response.

### Materials & experimental systems

| n/a | Involved in the study | |
|---|---|---|
| ☐ | ☒ | Antibodies |
| ☐ | ☒ | Eukaryotic cell lines |
| ☒ | ☐ | Palaeontology and archaeology |
| ☐ | ☒ | Animals and other organisms |
| ☒ | ☐ | Clinical data |
| ☒ | ☐ | Dual use research of concern |
| ☒ | ☐ | Plants |

### Methods

| n/a | Involved in the study | |
|---|---|---|
| ☐ | ☒ | ChIP-seq |
| ☒ | ☐ | Flow cytometry |
| ☒ | ☐ | MRI-based neuroimaging |

## Antibodies

| | |
|---|---|
| Antibodies used | GYS1 (Proteintech, Cat# 10566-1-AP, polyclonal, WB 1:1000); |

**Antibodies used**

GYS2 (Proteintech, Cat# 22371-1-AP, polyclonal, WB 1:1000);
PYGL (Proteintech, Cat# 15851-1-AP, polyclonal, WB 1:1000);
PYGM (Proteintech, Cat# 19716-1-AP, polyclonal, WB 1:1000);
AGL (Proteintech, Cat# 16582-1-AP, polyclonal, WB 1:1000);
DIO2 (Proteintech, Cat# 26513-1-AP, polyclonal, WB 1:500–1:1000);
GFP (Proteintech, Cat# 50430-2-AP, polyclonal, WB 1:2000–1:5000).

CST antibodies included
Glycogen Synthase (GYS1/GYS2) (Cell Signaling Technology, Cat# 3886, clone 15B1, WB 1:1000);
Phospho-HSL (Ser563) (CST, Cat# 4139, WB 1:1000);
Phospho-HSL (Ser660) (CST, Cat# 45804, WB 1:1000);
HSL (CST, Cat# 4107, WB 1:1000);
p38α MAPK (CST, Cat# 9218, WB 1:1000);
Phospho-p38 MAPK (Thr180/Tyr182) (CST, Cat# 9211, WB 1:1000);
α-Tubulin (CST, Cat# 3873, clone DM1A, WB 1:5000);
β-Actin (CST, Cat# 4970, clone 13E5, WB 1:5000);
ERRα (CST, Cat# 13826, clone E1G1J, WB 1:1000).

Additional antibodies were PGC1α (Sigma, Cat# AB3242, polyclonal, WB 1:1000), UCP1 (Abcam, Cat# ab209483, clone EPR20382, WB 1:1000), ERRγ (Abcam, Cat# ab219547, clone EPR20331, WB 1:1000), and PGC1β (Abcam, Cat# ab176328, clone EPR12342, WB 1:1000).ERR  antibody was generated by Dr. Ronald Evans laboratory.

**Validation**

All antibodies were used according to the manufacturers' recommendations. All the companies provide quality certificates for the antibodies used in the study. Each antibody was first tested with different dilutions, and optimal dilution ratios used in the study are stated in the methods section.

## Eukaryotic cell lines

Policy information about cell lines and Sex and Gender in Research

**Cell line source(s)**

HEK293T were purchased from the American Type Culture Collection (ATCC; https://www.atcc.org:443/)

**Authentication**

Cell line used were authenticated.

**Mycoplasma contamination**

Cells were routinely tested for Mycoplasma, and we confirm that every cell used in this study was found negative.

**Commonly misidentified lines**
(See ICLAC register)

No commonly misidentified cell lines were used.

## Animals and other research organisms

Policy information about studies involving animals; ARRIVE guidelines recommended for reporting animal research, and Sex and Gender in Research

**Laboratory animals**

C57BL/6J (strain no. 000664), PGC1  fl/fl (strain no. 009666), and Adipoq-Cre (strain no. 028020), Rosa26-Cas9 knockin on B6J (strain no. 026179) mice were obtained from The Jackson Laboratory. PGC1  fl/fl mice were crossed with Adipoq-Cre mice to generate adipocyte-specific PGC1  knockout mice. Esrra (ERR ) single knockout (ERR -AKO) and Esrra/Esrrb/Esrrg triple knockout (ERRs-AKO) mice were provided by Dr. Ronald Evans, all mice were maintained on a C57BL/6J background 67. All animal experiments were conducted in accordance with protocols approved by UCSD IACUC and were in compliance with the relevant ethical regulations regarding animal research.

**Wild animals**

The study did not involve wild animals.

**Reporting on sex**

ONLY male mice were used for experiment in this study.

**Field-collected samples**

Male mice aged 8 -12 weeks were maintained on standard chow (7912, Teklad) under specific pathogen-free (SPF) conditions with 12 h:12 h light–dark cycles and ad libitum access to food and water.

**Ethics oversight**

The animal experiments that were conducted as part of this research were completed in accordance with the guidelines provided by the UCSD IACUC

Note that full information on the approval of the study protocol must also be provided in the manuscript.

## Plants

| | |
|---|---|
| Seed stocks | N/A |
| Novel plant genotypes | N/A |
| Authentication | N/A |

## ChIP-seq

### Data deposition

☒ Confirm that both raw and final processed data have been deposited in a public database such as GEO.

☒ Confirm that you have deposited or provided access to graph files (e.g. BED files) for the called peaks.

| | |
|---|---|
| Data access links<br>*May remain private before publication.* | https://www.ncbi.nlm.nih.gov/sra/PRJNA1260692 |
| Files in database submission | ATAC-seq :ERRs-TKO-CL ,ERRs-TWT-CL,PGC1a-AKO-CL, PGC1a-WT-CL, ChIP-seq:ERRa in ERRs-TKO-CL ,ERRa in ERRs-TWT-CL |
| Genome browser session<br>(e.g. UCSC) | UCSC |

### Methodology

| | |
|---|---|
| Replicates | Two independent biological replicates were performed for each ChIP–seq experiment, using chromatin isolated from separate adipocyte differentiations (or mouse iWAT samples). Replicates showed highly consistent peak profiles and correlation (Pearson r > 0.9), and were used for downstream analysis. |
| Sequencing depth | 40M reads |
| Antibodies | ERRα (E1G1J) Rabbit mAb #13826 |
| Peak calling parameters | Peaks were called using MACS2 (v2.2.7.1) with default parameters and a q-value cutoff of 0.01, using input DNA as control. For transcription factors ERRα, narrow peaks were identified (–nomodel, –extsize 147). |
| Data quality | Sequencing quality was assessed using FastQC (v0.11.9). All libraries showed >90% of bases with Phred quality score ≥30. Reads were aligned uniquely to the mouse mm10 genome using Bowtie2 (v2.4.5) and low-quality or duplicate reads were removed with SAMtools (v1.15) and Picard (v2.26.10). Each sample yielded ~40 million paired-end reads. Biological replicates exhibited high reproducibility (Pearson correlation > 0.9) and consistent peak enrichment across replicates. |
| Software | Sequencing reads were aligned to the mouse reference genome (mm10) using Bowtie2 (v2.4.5) with local alignment mode and the --no-unal option. The resulting SAM files were converted to BAM format using samtools view (v1.15) and subsequently sorted with samtools sort. Duplicated reads were removed using Picard (v2.26.10)'s MarkDuplicates function. |
